# Upregulation of Glutaminyl Cyclase Contributes to ERS-Induced Apoptosis in PC12 Cells

**DOI:** 10.1155/2022/4154697

**Published:** 2022-11-28

**Authors:** Qi Shang, Xi Yu, Na Ouyang, Pan Xu, Xiaojie Chen, Yinan Wang, Chenyang Li, Xiaojuan Wang, Xifeng Lu, Chenshu Xu, Haiqiang Wu

**Affiliations:** ^1^School of Basic Medical Sciences, Health Science Center, Shenzhen University, Shenzhen, China; ^2^School of Pharmaceutical Sciences, Health Science Center, Shenzhen University, Shenzhen, China

## Abstract

Glutaminyl cyclase (QC) is responsible for converting the N-terminal glutaminyl and glutamyl of the proteins into pyroglutamate (pE) through cyclization. It has been confirmed that QC catalyzes the formation of neurotoxic pE-modified A*β* in the brain of AD patients. But the effects of upregulated QC in diverse diseases have not been much clear until recently. Here, RNA sequencing was applied to identify differentially expressed genes (DEGs) in PC12 cells with QC overexpressing or knockdown. A total of 697 DEGs were identified in QC overexpressing cells while only 77 in QC knockdown cells. Multiple bioinformatic approaches revealed that the DEGs in QC overexpressing group were enriched in endoplasmic reticulum stress (ERS) related signaling pathways. The gene expression patterns of 23 DEGs were confirmed by RT-qPCR, in which the genes related to ERS showed the highest consistency. We also revealed the protein levels of GRP78, PERK, CHOP, and PARP-1, and caspase family was significantly upregulated by overexpressing QC. Moreover, overexpressing QC significantly increased apoptosis of PC12 cells in a time dependent manner. However, no significant alteration was observed in QC knockdown cells. Therefore, our study indicated that upregulated QC could induce ERS and apoptosis, which consequently trigger diseases by catalyzing the generation of pE-modified mediators.

## 1. Introduction

Glutaminyl cyclase (QC), also known as glutaminyl-peptide cyclotransferase, was first isolated from the latex of Carica papaya, and later, its presence has been confirmed in a wide range of pro and eukaryotes [1, 2]. In human, QC is mainly expressed in neuronal tissues and catalyzes the pyroglutamate (pE) formation at the N-termini of newly formed peptides, which is necessary for maturation and physiological activities of hormones, cytokines, and enzymes [3–5].

Elevated QC expression has been observed in a variety of diseases, including Alzheimer's disease (AD), Huntington's disease, melanomas, thyroid carcinomas, accelerated atherosclerosis, and septic arthritics [6]. Moreover, QC expression positively correlates with the severity of the diseases [7, 8], indicating that QC plays an important role in the development of these diseases. Worthy to notice, pE-A*β*s and pE-CCL2, whose formation was catalyzed by QC, play critical roles in initiating and promoting the development of AD and early-stage inflammation [6]. Moreover, elevated QC expression can be detected in peripheral blood mononuclear cells of early-stage AD patients, when other AD-related biomarkers are still absent [9]. Based on these discoveries, inhibition of QC has therefore been proposed as a novel strategy in treating AD, leading to the *in vitro* and *in vivo* investigation of a series of QC inhibitors [10–14]. Therefore, it would be noteworthy to understand the effects of upregulated QC on inducing diseases. However, few reports have been focused on the effects until recently.

The endoplasmic reticulum stress- (ERS-) induced apoptosis has been implicated in inducing multiple diseases [15]. The endoplasmic reticulum (ER) is a fundamental organelle contributing to the protein quality control and cellular homeostasis. It is responsible for protein synthesis, translation, modification, and secretion in eukaryotic cells. The glucose regulated protein 78 (GRP78), also termed as binding immunoglobulin protein (Bip), is the molecular chaperone of ER and serves as a specific marker of endoplasmic reticulum stress (ERS) [16]. Under physiological condition, GRP78 is bound to three types of downstream type-I transmembrane proteins, including PKR-like ER kinase (PERK), inositol requiring enzyme 1 (IRE1), and activating transcription factor 6 (ATF6), and keep them in an inactive state. Under abnormal condition, the misfolded proteins accumulate in the lumen of the ER, inducing ER dysfunction and ERS [17]. When a large number of abnormal proteins accumulate in the ER, GRP78 is cleaved from these response proteins, which are then activated, and ERS is induced [18]. Subsequently, the cellular apoptosis is triggered mainly by increasing the transcription level of the proapoptotic transcription factor C/EBP homologous protein (CHOP), another contemporary and biomarker reflecting ERS [19]. ERS may deliver a potential signal for upregulated QC to induce diseases because overloaded pE-modified peptides or proteins usually act as misfolded or abnormal proteins during the development of diseases.

Apoptosis plays a critical role in the development and homeostasis of organisms [20]. Either excessive or insufficient apoptosis can results in severe pathological consequences. As we know, the caspase family has been well established to be involved in typical apoptosis. Normally, Caspase-3 is the ultimate effector for stimulating cellular apoptosis [21]. Cleaved caspase-3, the active form of caspase-3, is the main cleavage enzyme to promote apoptosis [22, 23]. In the process of apoptosis, the DNA damage sensor PARP-1, which has been applied as one of the most widely used diagnostic techniques for the detection of apoptosis in many cell types, can be cleaved by caspase-3 and involved in DNA damage and repair [24]. Interestingly, accumulating evidences have revealed that apoptosis induced by ERS plays crucial roles in the initiation of multiple diseases, including neurological diseases, cancers, diabetes, cardiovascular diseases, etc [15]. Therefore, the evaluation of ERS-induced apoptosis could be helpful to understand the effects of upregulated QC.

In the current study, we constructed PC12 cellular models by transducing cells with viruses overexpressing or knockdown QC. RNA-seq was then applied and DEGs were identified. Gene ontology (GO), Kyoto Encyclopedia of Genes and Genomics (KEGG) pathway, and protein-protein interaction (PPI) analyses were performed to reveal the DEGs related significant signaling pathways. Then, we investigated the role of QC in regulating DEGs and related pathways by determining the levels of gene and protein using RT-qPCR, western blot, and flow cytometry.

## 2. Materials and Methods

### 2.1. Recombinant Adenoviruses

Adenoviruses carrying the full-length QC gene were constructed by Shenggong Bioengineering Technology Limited (Shanghai, China). The overexpression of QC and its control (QC-OE and Ctrl-OE) were driven by cytomegalovirus (CMV) promoter in vector Psb50. QC knockdown and its control (QC-KD and Ctrl-KD) were constructed with a retrovirus vector pADV-U6-shRNA.

### 2.2. Cell Culture and Treatment

PC12 cells were cultured in 1 × DMEM (gibco, Cat.No. 11995065) supplemented with 8% Fetal Bovine Serum (FBS, gibco, Cat.No. 10100147C) and 2% Horse Serum (HS, gibco, Cat.No. 26050070) at 37°C in a humidity-controlled 5% CO_2_ cell culture incubator. PC12 cells were split with 1 × trypsin into 6-well culture dishes and allowed to adhere overnight before transduction by viruses for 0-60 h at 50 MOI.

### 2.3. RNA-Sequencing Analysis

Four groups of RNA samples were collected from PC12 cells after transduction for 48 h. Library construction and sequencing were performed with BGISEQ-500 by the Beijing Genomic Institution (http://www.genomic.org.cn, BGI, Shenzhen, China). Clean tags were mapped to the reference genome and genes available in the NCBI. For gene expression analysis, the matched reads were calculated and then normalized to RPKM using RESM software. The significance of differential gene expression was confirmed with the BGI bioinformatics service using the combination of the absolute value of log2 − Ratio ≥ 1 and *P* value≤0.001 in this study. Gene ontology (GO) and pathway annotation and enrichment analyses were based on the NCBI COG (http://www.ncbi.nlm.nih.gov/COG/), GO database (http://www.geneontology.org/), and Kyoto Encyclopedia of Genes and Genomes (KEGG) pathway database (https://www.genome.jp/kegg/). The software Cluster and Java Treeview were used for hierarchical cluster analysis of gene expression patterns.

### 2.4. RNA Extraction and RT-qPCR Verification

Quantitative real-time polymerase chain reaction (RT-qPCR) analysis was performed to validate the alteration in levels of DEGs revealed by RNA-seq analysis. Basically, total RNA was extracted from cells using HiPure Total RNA Mini Kit (Magen, Cat.No. R4111-02) and reverse transcribed into cDNA using the Primer ScriptTM RT Reagent Kit (TaKaRa, Cat.No. RR047Q). The prepared cDNA was then subjected to quantitative PCR analysis using TB Green® Premix Ex TaqTM (Tli RNaseH Plus) kit (TaKaRa, Cat.No. RR420Q) in a qTOWER 3G Cycler system (Analytik Jena AG, Germany). The sequences of the primers are presented in Table 1. The cycling condition was 95°C for 30 s, followed by 40 cycles at 95°C for 5 s, and 60°C for 30 s. The fold induction was calculated as described previously [25].

### 2.5. Western Blot Analyses

Total proteins were extracted from PC12 cells using RIPA buffer (Absin Bioscience Inc, Shanghai, China), and protein concentration was determined with TaKaRa BCA Protein Assay Kit (TaKaRa, Cat.No. T9300A). Equal amounts of proteins were separated by 12.5% SDS-PAGE and transferred to polyvinylidene fluoride membranes that were probed with primary antibodies including QC (Abcam, Cat.No. 14220 T), GRP78 (Abcam, Cat.No.ab21685), PERK (Abcam, Cat.No. ab229912), CHOP (Proteintech, Cat.No. 15204-1-AP), caspase12 and cleaved caspase12 (Abcam, Cat.No. 62484), caspase3 and cleaved caspase 3 (CST, Cat.No. 14220 T), cleaved PARP1 (Abcam, Cat.No. ab32064), and GAPDH (CST, Cat.No. 2118S). Immunodetection was performed using the ClarityTM western ECL substrate (Bio-RED, Cat.No. 170-5061) and quantified with ImageJ software (ImageJ v.1.48 V).

### 2.6. Flow Cytometry

After transduced with viruses for 24-48 h, PC12 cells were harvested, washed, and stained with Annexin-V APC/7-AAD cellular apoptosis assay kit (Elabscience, Cat.No. E-CK-A218) according to the manufacturer's instructions. Briefly, cells were washed twice with PBS, collected in flowcytometric polystyrene tubes at 1 × 10^5^ cells/tube, and resuspended in 500 *μ*l Annexin-binding buffer. Subsequently, 2.5 *μ*l of Annexin V-APC was added and mixed well with the samples. After adding 2.5 *μ*l of 7-AAD, samples were analyzed by Cytoflex flow cytometer (Beckman) and FlowJo Software (v10). Four subpopulations were identified: normal cells (Annexin V-APC−/7-AAD−), necrotic cells (Annexin V-APC−/7-AAD+), early apoptotic cells (Annexin V-APC+/7-AAD−), and late apoptotic cells (Annexin V-APC+/7-AAD+). Apoptosis index was calculated according to the total of early apoptotic and late apoptotic cells.

### 2.7. Statistical Analyses

Data are presented as mean ± SD. A *P* value<0.05 was considered statistically significant. All experiments were performed at least three times. The results were analyzed using one-way ANOVA with Tukey multiple comparison by SPSS 22.0 software.

## 3. Results

### 3.1. Identification of DEGs upon QC Overexpression or Knockdown

**Figure 1:**
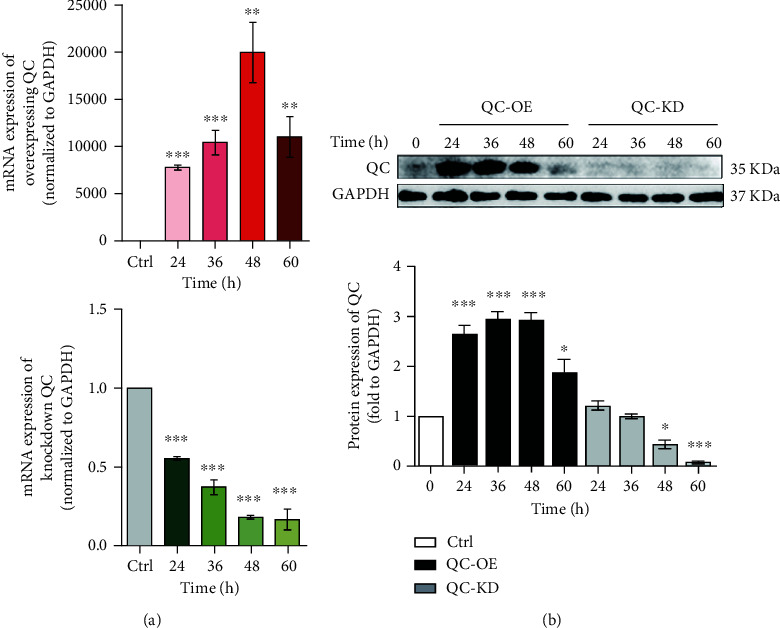
The establishment of PC12 cells with QC overexpression or knockdown. PC12 cells were infected with adenovirus with QC over-expression or retrovirus with QC knockdown for different hours as indicated. The mRNA (a) and protein (b) expressions of QC were then analyzed. Data are expressed as fold change over the control group. ^∗^*P* < 0.05, ^∗∗^*P* < 0.01, and ^∗∗∗^*P* < 0.001 indicate statistically significant effects compared to control group (*n* = 3).

Firstly, viruses carrying QC overexpression or knockdown genes were transduced into PC12 cells. RT-qPCR results showed that the mRNA levels of QC were significantly increased 24 h after transduction with QC overexpressing virus and peaked at 48 h. On the contrary, transduction with QC knockdown virus decreased the QC mRNA levels (Figure 1(a)). Similar results were obtained in the protein expression of QC (Figure 1(b)), indicating the successful establishment of the cellular models.

**Figure 2:**
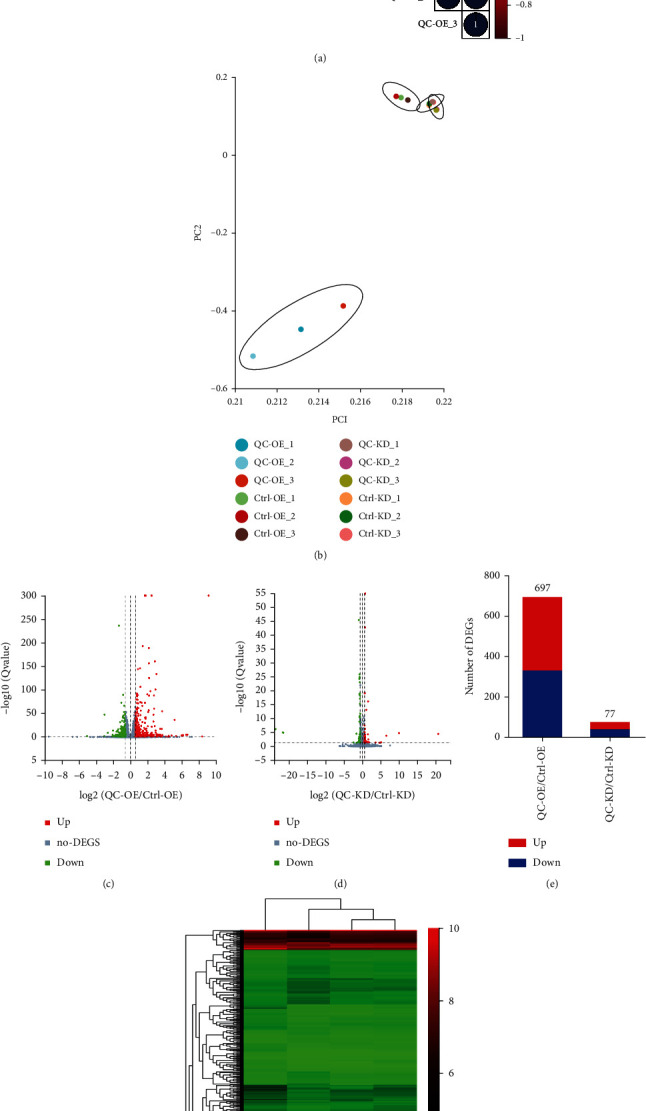
The bioinformatics profile of DEGs. (a) Heat map of correlation coefficients of relative gene expression levels. The number in each circle represents the correlation coefficient (*R* value). (b) Principal component analysis of RNA-seq data of four groups of samples. (c, d) The numbers of DEGs identified in groups of QC-OE vs. Ctrl-OE and QC-KD vs. Ctrl-KD. Volcano plots illustrated differentially regulated gene expression from RNA-seq analysis between groups of QC-OE vs. Ctrl-OE (c), and QC-KD vs. Ctrl-KD (d) in PC12 cells. (e) Bar chart of the numbers of DEGs identified in groups of QC-OE vs. Ctrl-OE and QC-KD vs. Ctrl-KD. (f) Heat map of DEGs showing hierarchical clustering of all changed DEGs in groups of QC-OE vs. Ctrl-OE and QC-KD vs. Ctrl-KD. The overall FPKM hierarchical clustering map was developed using log(value + 1); upregulated and downregulated genes are colored in red and green, respectively.

To study the global gene expression profiling regulated by QC, an unbiased high-throughput RNA-seq analysis was utilized. Correlation analysis heatmaps for each condition showed a strong correlation across replicates (Figure 2(a)), demonstrating that replicates within each group from the same experimental condition were similar and statistically close to each other. The normalized results between QC-KD, Ctrl-KD and Ctrl-OE groups exhibited a high correlation coefficient ranging from 0.980–1.000, suggesting the extremely similar normalization capabilities. However, QC-OE group showed relatively low correlation coefficients when compared with the other groups (0.919–0.956). Principal Components Analysis (PCA) was then performed to investigate the gene expression patterns. PCA using DEGs displayed a clear separation between QC-OE and the other groups (Figure 2(b)), indicating a significant role of overexpressed QC in regulating biological functions in PC12 cells. To investigate the different expression patterns of genes between groups of QC-OE vs. Ctrl-OE and QC-KD vs. Ctrl-KD, volcano plots were generated. A total of 697 DEGs were identified in groups of QC-OE vs. Ctrl-OE, with 364 (52.22%) genes being upregulated and 333 (47.78%) being downregulated. In contrast, only 77 DEGs were identified in groups of QC-KD vs. Ctrl-KD (Figures 2(c)–2(e)). The numbers of DEGs identified in each group were highly corresponding to their distances in PCA analysis. DEGs were also hierarchically clustered depending on the gene enrichment features of QC overexpressing or knockdown against their control groups. The most prominently upregulated genes consisted of members in Heat Shock Protein Family, including GRP78, hsp90b1, and hyou1 (Figure 2(f)).

### 3.2. GO and KEGG Pathway Analysis

**Figure 3:**
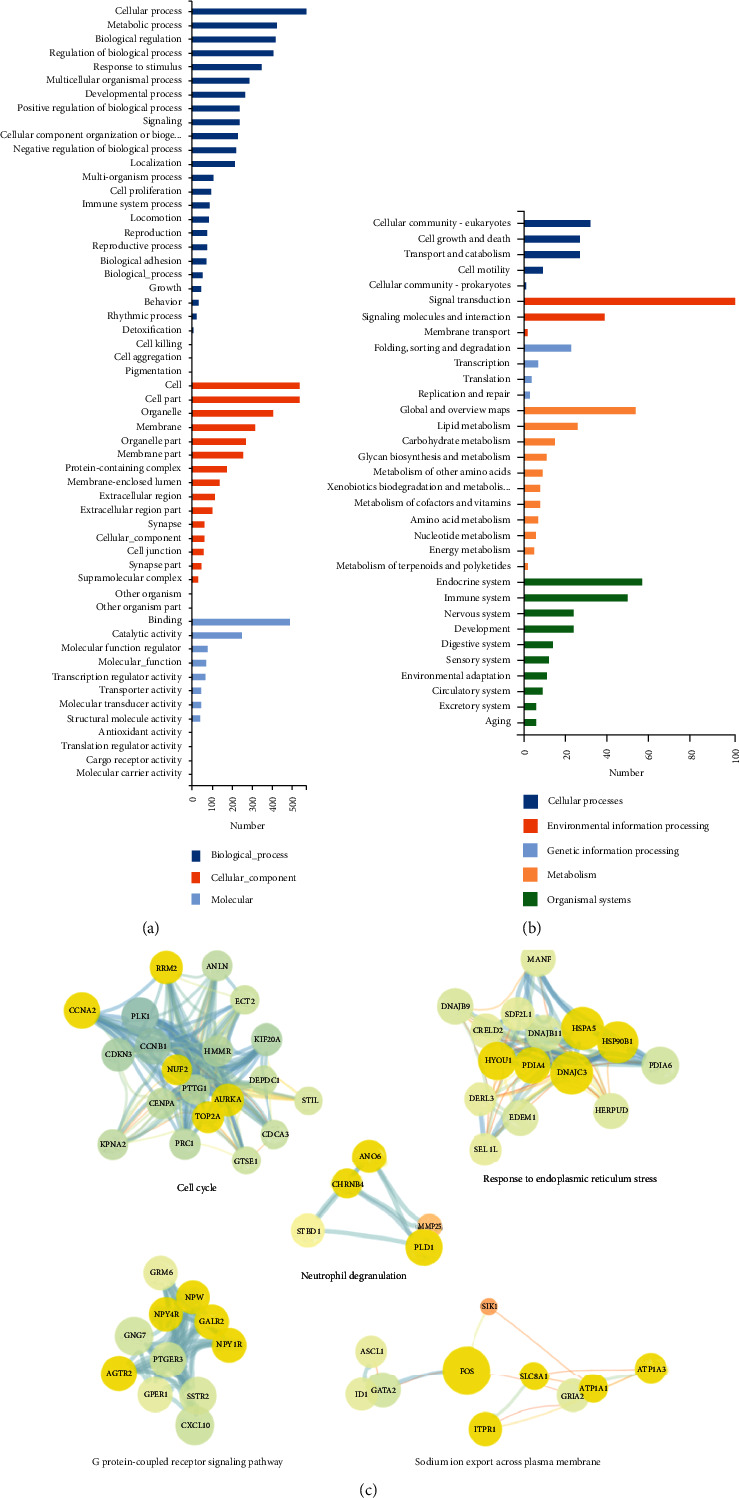
The GO, KEGG, and PPI network analyses of the DEGs. (a) GO analysis was conducted in groups of QC-OE vs. Ctrl-OE and covered three domains: cellular components, biological process, and molecular function. (b) KEGG analysis was conducted in groups of QC-OE vs. Ctrl-OE. (c) The online biological resource database Search Tool for the Retrieval of Interacting Genes/Proteins (STRING) 7 was used to identify the interactions between known and predicted proteins. The PPI network was constructed with a score > 0.408, and visualized by Cytoscape 3.7.2, a free software package for visualizing, modeling, and analyzing the integration of biomolecular interaction network with high-throughput expression data. The plug-in named Molecular Complex Detection (MCODE) was then used to filter the modules of the PPI network with parameters set as follows: *K* − core = 2, node score cutoff = 0.2, degree cutoff = 2, and Max depth up to 100. The top 5 modules are presented in (b). Node color represents the degrees of DEGs, in which brighter node colors correspond to higher degrees of DEGs. Node size represents the closeness centrality, in which the node size is proportional to its closeness centrality. Line thickness represents combined score, in which thicker lines indicate a closer relationship between the connected nodes.

We next performed gene ontology (GO) to identify more subtle alternations in overall pathways and functional sets of genes. On the basis of significant enrichment (FDR < 0.05), 27 biological processes were found differentially enriched, which were related to following processes such as cellular process, metabolic process, biological regulation, etc (Figure 3(a)). Under category of cellular components, we found that 17 GO terms were significantly enriched including cell, cell part, organelle, etc. Similarly, under category of molecular function, we found that 12 GO terms were significantly enriched including binding, catalytic activity, molecular function, etc.

The GO enrichment analysis of Biological Process demonstrated significantly affected categories of genes. Upregulated genes were significantly enriched in DNA integration, virion assembly, virus process, and neuron differentiation in response to QC overexpressing (Figure S1(a) and S1(b)), while downregulated genes were significantly enriched in cell adhesion, extracellular matrix organization, skeletal system development, and osteoblast differentiation.

In addition, the GO analysis of Cellular Component showed that upregulated genes were significantly enriched in endoplasmic reticulum lumen, endoplasmic reticulum chaperone complex, endoplasmic reticulum, and smooth endoplasmic reticulum upon QC overexpression (Figure S1(c) and S1(d)), while downregulated genes were significantly enriched in extracellular matrix, extracellular space, collagen-containing extracellular matrix, and extracellular region.

Moreover, GO analysis of Molecular Functions showed that upregulated genes were significantly enriched in RNA-directed DNA polymerase activity, RNA-DNA hybrid ribonuclease activity, and endonuclease activity upon QC overexpression, while downregulated genes were enriched in platelet-derived growth factor binding, extracellular matrix structural constituent, and calcium ion binding upon QC overexpression. The top 20 enriched gene sets of are listed in Figure S1(e) and S1(f).

To elucidate the biological functions of DEGs and interaction in cells, we performed Kyoto Encyclopedia of Genes and Genomics (KEGG) pathway analysis. The DEGs between QC overexpressing against control group were subjected to KEGG pathway enrichment analysis using the DNBSEQ platform. We identified 212 enriched KEGG pathways (*P* < 0.05) affected by QC overexpression, which were clustered into 5 groups including cellular processes, environmental information processing, genetic information processing, metabolism, and organismal systems. Some of the highly enriched pathways include cellular community, signal transduction, folding sorting and degradation, global and overview maps, endocrine system, etc., as shown in Figure 3(b). And, the upregulated DEGs were significantly enriched in signaling pathways such as protein process in endoplasmic reticulum, MAPK signaling pathway, and oocyte meiosis (Figures S2(a) and S2(b)), while the downregulated DEGs were enriched in ECM-receptor interaction, protein digestion and absorption, and arachidonic acid metabolism.

### 3.3. PPI Network Analysis of the DEGs

To explore the interactions among proteins encoded by the QC overexpression-related DEGs, the PPI network of the 697 DEGs was constructed, which contained 552 nodes and 1677 edges (Figure S3). Based on filtering the modules of the PPI network, the top five modules were selected. The DEGs in these modules were related to many significant signaling pathways, including cell cycle, response to endoplasmic reticulum stress, G protein-coupled receptor signaling pathway, neutrophil degranulation, and sodium ion export across plasma membrane (Figure 3(c)). These pathways could be supposed as the potential pathways regulated by the overexpressed QC in PC12 cells.

### 3.4. Identification of Hub Genes

**Figure 4:**
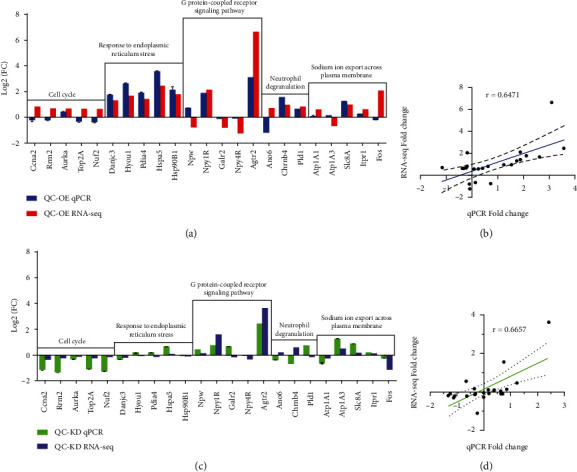
Validation of RNA-seq results with RT-qPCR in groups of QC-OE vs. Ctrl-OE and QC-KD vs. Ctrl-KD. Gene expressions of 23 selected genes were analyzed (a, c). Data are expressed as fold change over the control group. Correlation analysis was also performed between RNA-seq and RT-qPCR (b, d).

According to PPI network analysis, 23 DEGs (19 upregulated and 4 downregulated genes) from the top five modules were selected to evaluate furtherly. The RT-qPCR and RNA-seq results of these genes are shown in Figures 4(a) and 4(c). It was clear that the gene expression patterns determined by RT-qPCR were highly consistent with the data determined by RNA-seq. Correlation analysis of results obtained from these two methods showed that these results were significantly correlated (*r* = 0.6471^∗∗^, *r* = 0.6657^∗∗^, Figures 4(b) and 4(d)), supporting the data obtained using RNA-seq analysis for the identification of key genes in response to QC overexpression or knockdown in PC12 cells. Among the top five modules, more importantly, the gene expression profiles in the module related to response to ERS showed the highest consistency, which included Danjc3, Hyou1, Pdia4, Hspa5, and Hsp90B1. Observation here showed that overexpression of QC upregulated the RNA expression of these genes in both RNA-seq and RT-qPCR analysis, while no much change was detected upon QC knockdown. These results indicated a potential role of QC in activating ERS in PC12 cells.

### 3.5. Regulation of ERS

**Figure 5:**
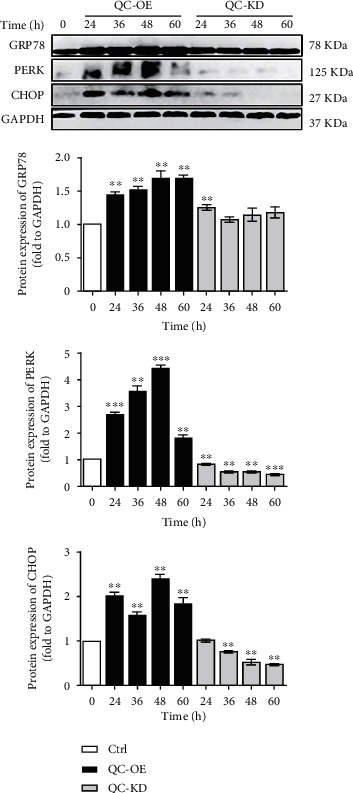
Effect of QC overexpression or knockdown on protein expression of ERS-related genes in PC12 cells. PC12 cells were infected with adenovirus with QC overexpression or retrovirus with QC knockdown for different hours as indicated. The protein expressions of GRP78, PERK and CHOP were then analyzed. Data are expressed as fold change over the control group. ^∗^*P* < 0.05, ^∗∗^*P* < 0.01, and ^∗∗∗^*P* < 0.001 indicate statistically significant effects compared to control group (*n* = 3).

As we mentioned above, RNA-seq and RT-qPCR analysis revealed that the DEGs in QC overexpressing group were highly enriched in endoplasmic reticulum and ERS related signaling pathways, and overexpression of QC upregulated the RNA levels of selected DEGs involved in ERS. Therefore, we investigated the role of QC in regulating ERS by determining the expression of proteins involved in ERS in QC overexpressing or knockdown cells, such as GRP78, PERK, and CHOP. Our results showed that the protein levels of GRP78, PERK, and CHOP were significantly increased in response to overexpression of QC (Figure 5). The protein expression of GRP78 was increased in a time dependent manner, while the protein expression of PERK was increased with a maximum induction at 48 h when compared to control group. However, QC knockdown exerted no effect on the protein expression of GRP78, while reduced the protein expression of PERK and CHOP in a time dependent manner. These results indicated that overexpression, but not knockdown of QC triggers the ERS in the PC12 cells, which may induce the subsequent cellular apoptosis in diseases.

### 3.6. The Involvement of QC in Apoptosis

**Figure 6:**
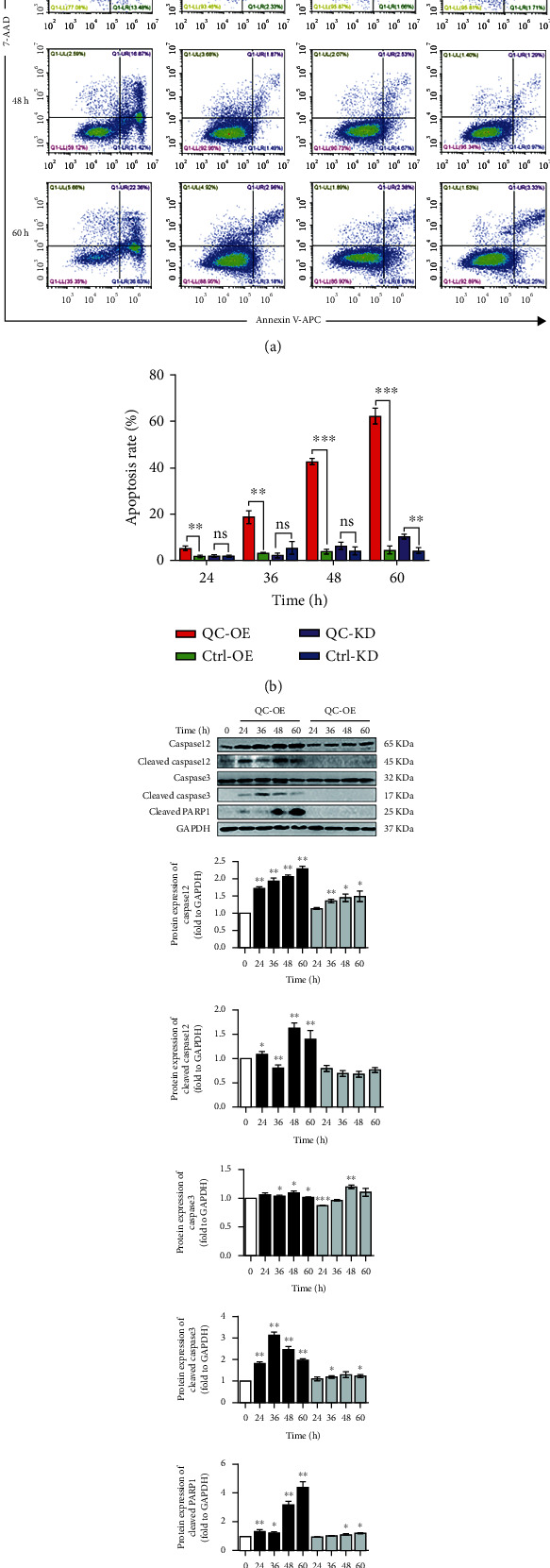
Effect of QC overexpression or knockdown on apoptosis in PC12 cells. PC12 cells were infected with adenovirus with QC overexpression or retrovirus with QC knockdown for different hours as indicated. The cellular apoptosis was detected by Flow cytometry and quantified using FlowJo software. The protein expressions of caspase12, cleaved caspase12, caspas3, cleaved caspase3, and cleaved PARP1 were then analyzed. Data are expressed as fold change over the control group. ^∗^*P* < 0.05, ^∗∗^*P* < 0.01, and ^∗∗∗^*P* < 0.001 indicate statistically significant effects compared to control group (*n* = 3).

ERS normally contributes to the initiation of apoptosis. In this research, we showed the regulation of ERS when QC was overexpressed or knockdown. Then, the effects of overexpressed or knockdown QC on apoptosis by inducing ERS were investigated. Flow cytometry using Annexin V-APC/7-AAD staining showed that the apoptosis of PC12 cells was significantly elevated in a time dependent manner with QC overexpression compared with the control group (Figures 6(a) and 6(b)). However, no much change was observed in QC knockdown cells. Western blotting analysis demonstrated that overexpressing QC significantly increased the expression of caspase12, cleaved caspase12, and cleaved PARP1 in a time dependent manner. Similar results were also observed in QC knockdown cells but to a much less extent. The expression of caspase3 was slightly increased at 36, 48, and 60 hours after QC overexpression, which, however, significantly increased the protein expression of cleaved caspase3. On the contrary, no much change was observed upon QC knockdown (Figure 6(c)). The above data collectively suggested the upregulation of QC in inducing apoptosis in PC12 cells, possibly through activating members of caspase family and PARP-1.

## 4. Discussion

The pE modification, mainly catalyzed by QC, is prevalent throughout nature and particularly important in humans for maturation of functional peptides and proteins. However, the upregulation of QC has been confirmed to be involved in multiple diseases and abnormal conditions including neurodegenerative diseases, cancers, inflammation, etc. by catalyzing the generation of pE-modified mediators such as pE-A*β* and pE-CCL2. But the mechanism in details through which upregulated QC induces diseases needs to be investigated deeply to improve the discovery and development of novel agents and therapeutics.

RNA-seq is an efficient technology for transcriptome analysis of gene expression. It produces a digital signal directly from the cDNA template and enables the detection of DEGs during biological process [6, 26, 27]. Its advantages include providing a low frequency of false-positive signals, highly reproducible results, and an exponential increase in sequencing capacity at a relatively low cost. Here, RNA-seq analysis demonstrated that a total of 697 DEGs were identified in QC overexpressing cells. While, only 77 DEGs were identified in QC knockdown cells. Similarly, research demonstrated that QC depletion exerts no significant abnormalities in QC knock-out mice, as adult homozygous QC knock-out mice are fertile and their motor function, cognition, general activity, and ingestion behavior are indistinguishable from wild type mice [28]. Therefore, the effects of QC overexpressing were focused here.

The GO analysis of cellular component showed that upregulated genes were significantly enriched in endoplasmic reticulum, and KEGG pathway enrichment analysis revealed that the upregulated DEGs were significantly enriched in signaling pathways such as protein process in endoplasmic reticulum. Moreover, the top five modules selected from PPI network and analyzed in terms of enriched pathways also included response to ERS. Therefore, we further investigated the potential role of QC in regulating ERS.

ERS is a cellular defense against stress in the body, which can induce unfolded protein response (UPR) to promote the protein folding mechanism and protect cells. However, strong and continuous stress may induce apoptosis, interferes with cellular homeostasis, and causes functional disorders in cells or tissues. Multiple studies have revealed the close association between ERS-induced apoptosis and diverse diseases, such as cancers, neurological diseases, cardiovascular diseases, etc. [17]. ERS requires the involvement of three ER transmembrane proteins: PERK, ATF6, and IRE1, which are bound to the glucose-regulated protein molecular chaperones GRP78 under nonactive state [16, 18]. Upon ERS, unfolded or misfolded proteins will cause the dissociation of GRP7 from these receptor molecules, induce the transduction of their downstream signals and expression of related genes, including CHOP, and further trigger cellular apoptosis [19]. Our study showed that the protein levels of GRP78, PERK, and CHOP were significantly upregulated when QC was overexpressed, with a maximum induction at 48 h. However, no effect was observed in QC knockdown cells. So, upregulated QC may induce ERS by catalyzing the generation of pE-modified proteins which contribute to continuous stress and functional disorders consequently.

Imbalance of apoptosis usually acts as the main inducer for the development of diseases, and abnormal apoptosis is always triggered by ERS. In the current study, we further investigated the apoptosis in PC12 cells. Data obtained here showed that overexpressing QC also increased the expression of the apoptosis markers, including caspase12, cleaved caspase12, caspase3, cleaved caspase3, and cleaved PARP1. These data indicated a potential role of QC in regulating ERS-induced apoptosis.

**Figure 7:**
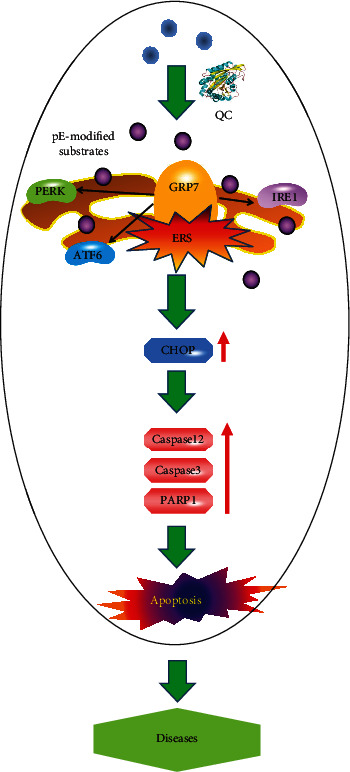
A working model proposed regarding the potential role of upregulated QC in inducing cellular apoptosis through regulating ERS in multiple diseases.

The pE-modification catalyzed by QC increases the hydrophobicity and stability of the substrates, the pH-dependent solubility profiles of which are also changed. Because of the presence of pE, the pE-modified mediators exhibit different physical and chemical properties and biofunctions, e.g., pE-A*β*s. Compared with A*β*, pE-A*β*s aggregate more rapidly, confer enhanced stability and proteolytic resistance, and seed further A*β* aggregation. Meanwhile, pE-A*β* can drive the downstream toxicity cascade to destroy the plasticity of synapses and induce the death of neurons through the formation of much stronger neurotoxic oligomers and ‘infection' of Tau, ROS, Ca^2+^ and other pathways [29–31]. Consequently, pE-A*β*s exhibit greater neurotoxicity compared with A*β*s and contribute to the dysfunction of neurons and initiation of AD. Therefore, it is reasonable to propose a hypothesis: the abnormal upregulated QC promotes the pE-modification of the critical elements, the solubility and toxicity of which are enhanced significantly. Failing to go through regular protein degradation, these pE-modified substrates tend to accumulate in the ER, induce ERS, and damage the normal functions of ER. Then, abnormal apoptosis is induced by ERS, and diseases are triggered consequently (Figure 7). Thus, it is of particular interest to identify the potential pE-modified targets of QC, which may further elucidate the crucial role of QC in development of diseases. Moreover, QC is supposed as a novel target for the treatment of diseases.

## 5. Conclusion

In the current study, we explore the potential mechanism of upregulated QC to trigger diseases in PC12 cells for the first time. Our study indicated the potential role of QC over-expression in regulating ERS by catalyzing the generation of pE-modified mediators, which subsequently induces cellular apoptosis and diseases. Data obtained here could contribute to the understanding of the effects of upregulated QC in diseases.

## Figures and Tables

**Table 1:** Primer sequences used for RT-qPCR.

Gene	5′-3′ sequence
Ccna2	
Forward	TTCACAGCCAAATGCAGGGTCTC
Reverse	GAGGCAGCCAGACATCACTAACAG
Rrm2	
Forward	CACCCACTAAGCCCAGCATTGAG
Reverse	GGTCCACCTCCTCGGCAGTC
Aurka	
Forward	GGTATTTCCATGACGCCACCAGAG
Reverse	AGCATTCGCCAACTCCGTGATG
Top2a	
Forward	AGCAGAAGGTCCCAGAAGAAGAGG
Reverse	AGGTAGTTGAAGGTCGGTCCAGAC
Nuf2a	
Forward	AGAAGACGACGGAGGAGTGCTG
Reverse	TCAGGGCGGCATTTTGAACATCC
Dnajc3	
Forward	CTCGCCGAAGCAGAGGATGA
Reverse	GCGCTGTCAAAGGCATCGAG
Hyou1	
Forward	TCGTATGGCTGGCCTCAAGG
Reverse	TGCCCGAGCCCATGTCATAG
Pdia4	
Forward	GCTAAGCGGTACAGCAAGCG
Reverse	TCAGGGAAGTCCTTGGCCAC
Hspa5	
Forward	CGGAGGAGGAGGACAAGAAGGAG
Reverse	ATACGACGGTGTGATGCGGTTG
Hsp90b1	
Forward	AGAGTCTCCCTGTGCCCTTGTG
Reverse	CCGTCTGGTATGCTTGTGCCTTC
Npw	
Forward	CTGCTGCTTCTGCTCTTGCTACC
Reverse	GCACGACCCACTGTGTGATAGC
Npy1r	
Forward	TGCGGCGTTCAAGGACAAGTATG
Reverse	GCAGCACCAGAAGAAGAGTCGTG
Galr2	
Forward	GTTTGGTCGCTTCCCGCTCAC
Reverse	GGTTGACACAGGAGTTGGCATAGG
Npy4r	
Forward	CTTGTTTCCTCTCCTTGCCCTTCC
Reverse	TGTAGATGAGGCGGTGGTGATCC
Agtr2	
Forward	GCTGCTGTTGTGTTGGCATTCATC
Reverse	ATACCCATCCAGGTCAGAGCATCC
Ano6	
Forward	GCTGCCTCCTTGAACTGACTACAC
Reverse	CATAACCCACGGGAGCAGAACTTC
Chrmb4	
Forward	CCAGGACGGAGGACGGTGAAC
Reverse	AGGATAGCCAGCGAGGTGATGAG
Pld1	
Forward	AACCGCTGGAGGCTGGACTG
Reverse	CCGCTTGGTGTACTCGCTGTTG
Atp1a1	
Forward	GCTCTTGCTGCTTTCCTGTCCTAC
Reverse	GCTTCCGCACCTCGTCATACAC
Atp1a3	
Forward	CGCACTGTCAACGACCTGGAAG
Reverse	CCACCACTATGCTCACGAAGAAGG
Slc8a	
Forward	CACCCAACACTGCCACCATAACC
Reverse	ATGATGCCAATGCTCTCGCTCAC
Itpr1	
Forward	CAAGGCGACAGTGACCGTGAAC
Reverse	TTGTGGGCTCTTTGGCTTTCTTCC
Fos	
Forward	AGACCATGTCAGGCGGCAGAG
Reverse	GTCAGCTCCCTCCTCCGATTCC
QC	
Forward	AGGCTGAGTGGGTCGTGGAAG
Reverse	TGGAGTCATAGTGGCAGGCAAGG
GAPDH (rat)	
Forward	GACATGCCGCCTGGAGAAAC
Reverse	AGCCCAGGATGCCCTTTAGT

## Data Availability

The data that support the findings of this study are available from the corresponding author upon reasonable request.

## Supplementary Materials

Supplementary Materials Figure S1: GO pathway analysis of the DEGs. GO analysis was conducted in groups of QC-OE vs. QC-Ctrl and covered three domains: cellular components, biological process, and molecular function. The top 20 biological processes in which the upregulated (a) or downregulated (b) genes were significantly enriched. The top 20 cellular components in which the upregulated (c) or downregulated (d) genes were significantly enriched. The top 20 molecular functions in which the upregulated (e) or downregulated (f) genes were significantly enriched. Figure S2: KEGG pathway analysis of the DEGs. KEGG analysis was conducted in groups of QC-OE vs. QC-Ctrl. The top 20 signaling pathways in which the upregulated (a) or downregulated (b) genes were significantly enriched. Figure S3: the PPI network of the DEGs. The online biological resource database Search Tool for the Retrieval of Interacting Genes/Proteins (STRING) 7 was used to identify the interactions between known and predicted proteins. The PPI network was constructed with a score > 0.408, and visualized by Cytoscape 3.7.2, a free software package for visualizing, modeling, and analyzing the integration of biomolecular interaction network with high-throughput expression data. The plug-in named Molecular Complex Detection (MCODE) was then used to filter the modules of the PPI network with parameters set as follows: *K* − core = 2, node score cutoff = 0.2, degree cutoff = 2, and Max depth up to 100. 552 nodes and 1677 edges are displayed (a). Node color represents the degrees of DEGs, in which brighter node colors correspond to higher degrees of DEGs. Node size represents the closeness centrality, in which the node size is proportional to its closeness centrality. Line thickness represents combined score, in which thicker lines indicate a closer relationship between the connected nodes.
