# Supplementary material for: Upregulation of Glutaminyl Cyclase Contributes to ERS-Induced Apoptosis in PC12 Cells
Source: Biomed Res Int. 2022 Nov 28;2022:4154697. doi: 10.1155/2022/4154697 (PMC9722295; doi:10.1155/2022/4154697)

**Figure S1.** GO pathway analysis of the DEGs. GO analysis was conducted in groups of QC-OE vs. QC-Ctrl., and covered three domains: cellular components, biological process, and molecular function. The top 20 biological processes in which the upregulated (A) or downregulated (B) genes were significantly enriched. The top 20 cellular components in which the upregulated (C) or downregulated (D) genes were significantly enriched. The top 20 molecular functions in which the upregulated (E) or downregulated (F) genes were significantly enriched.

**Figure S2.** KEGG pathway analysis of the DEGs. KEGG analysis was conducted in groups of QC-OE vs. QC-Ctrl. The top 20 signaling pathways in which the upregulated (A) or downregulated (B) genes were significantly enriched.

**Figure S3.** The PPI network of the DEGs. The online biological resource database Search Tool for the Retrieval of Interacting Genes/Proteins (STRING) 7 was used to identify the interactions between known and predicted proteins. The PPI network was constructed with a score > 0.408, and visualized by Cytoscape 3.7.2, a free software package for visualizing, modeling and analyzing the integration of biomolecular interaction network with high-throughput expression data. The plug-in named Molecular Complex Detection (MCODE) was then used to filter the modules of the PPI network with parameters set as follows: K-core = 2, node score cutoff = 0.2, degree cutoff = 2 and Max depth up to 100. 552 nodes and 1677 edges are displayed (A). Node color represents the degrees of DEGs, in which brighter node colors correspond to higher degrees of DEGs. Node size represents the closeness centrality, in which the node size is proportional to its closeness centrality. Line thickness represents combined score, in which thicker lines indicate a closer relationship between the connected nodes.
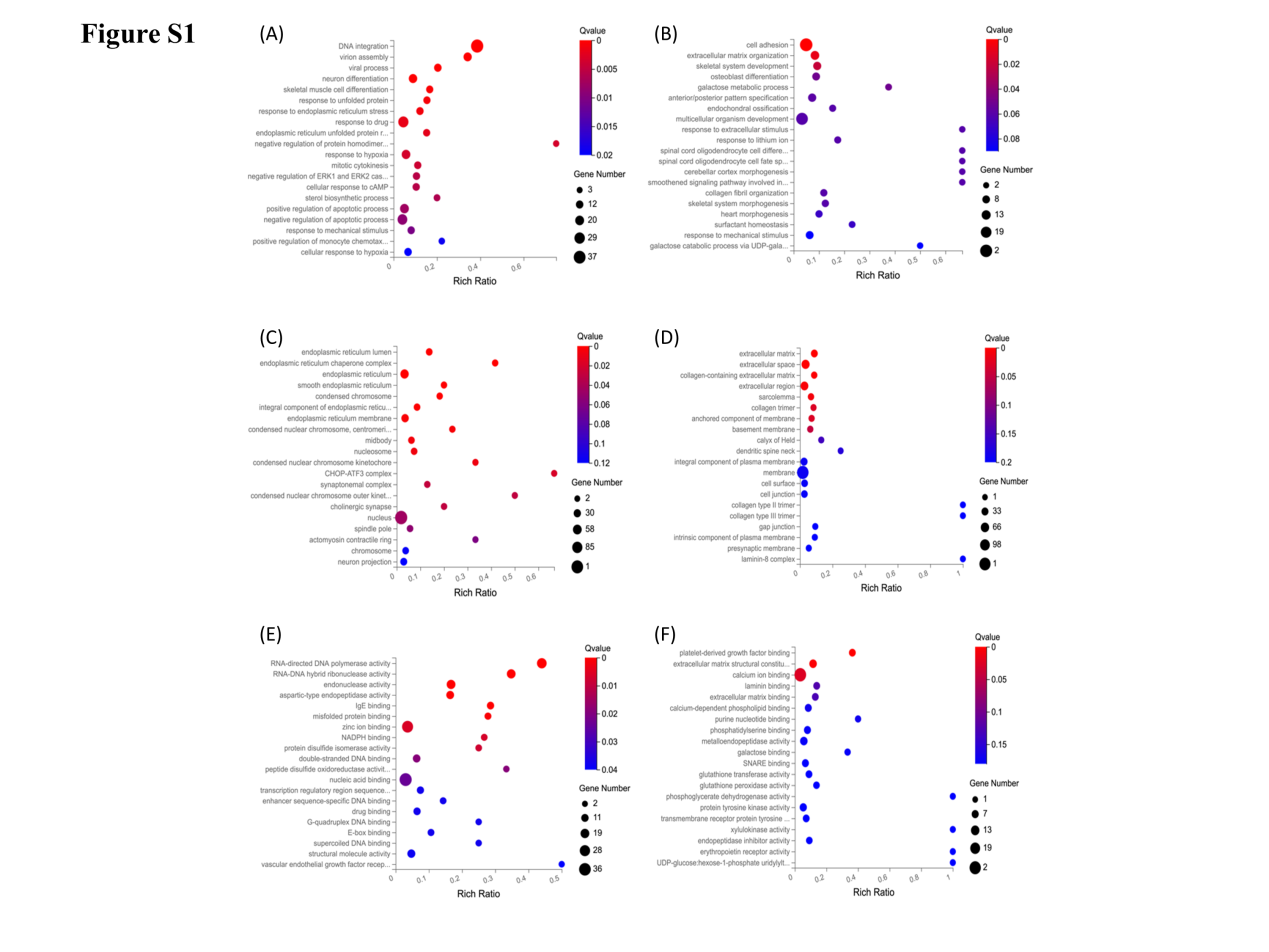


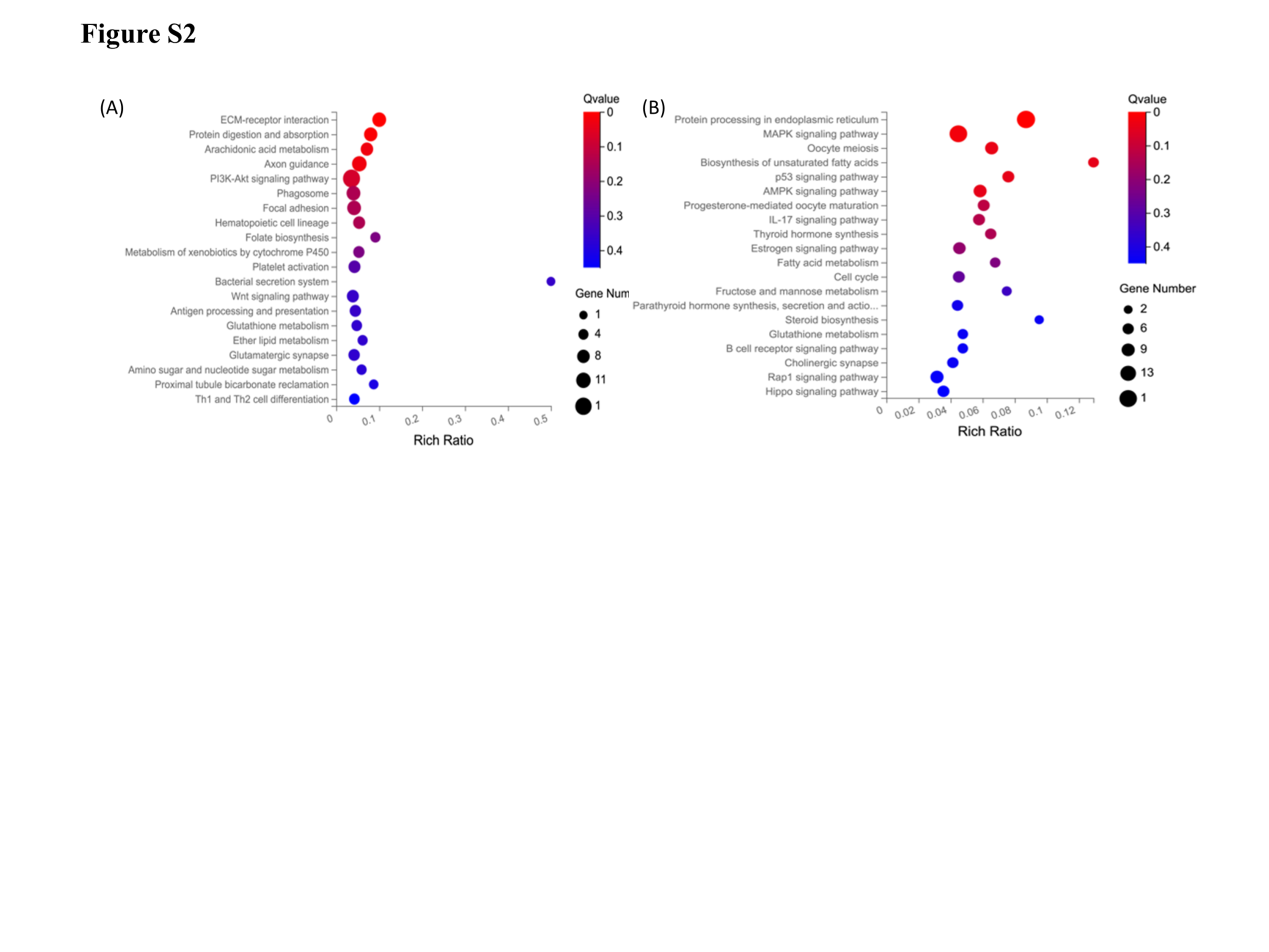


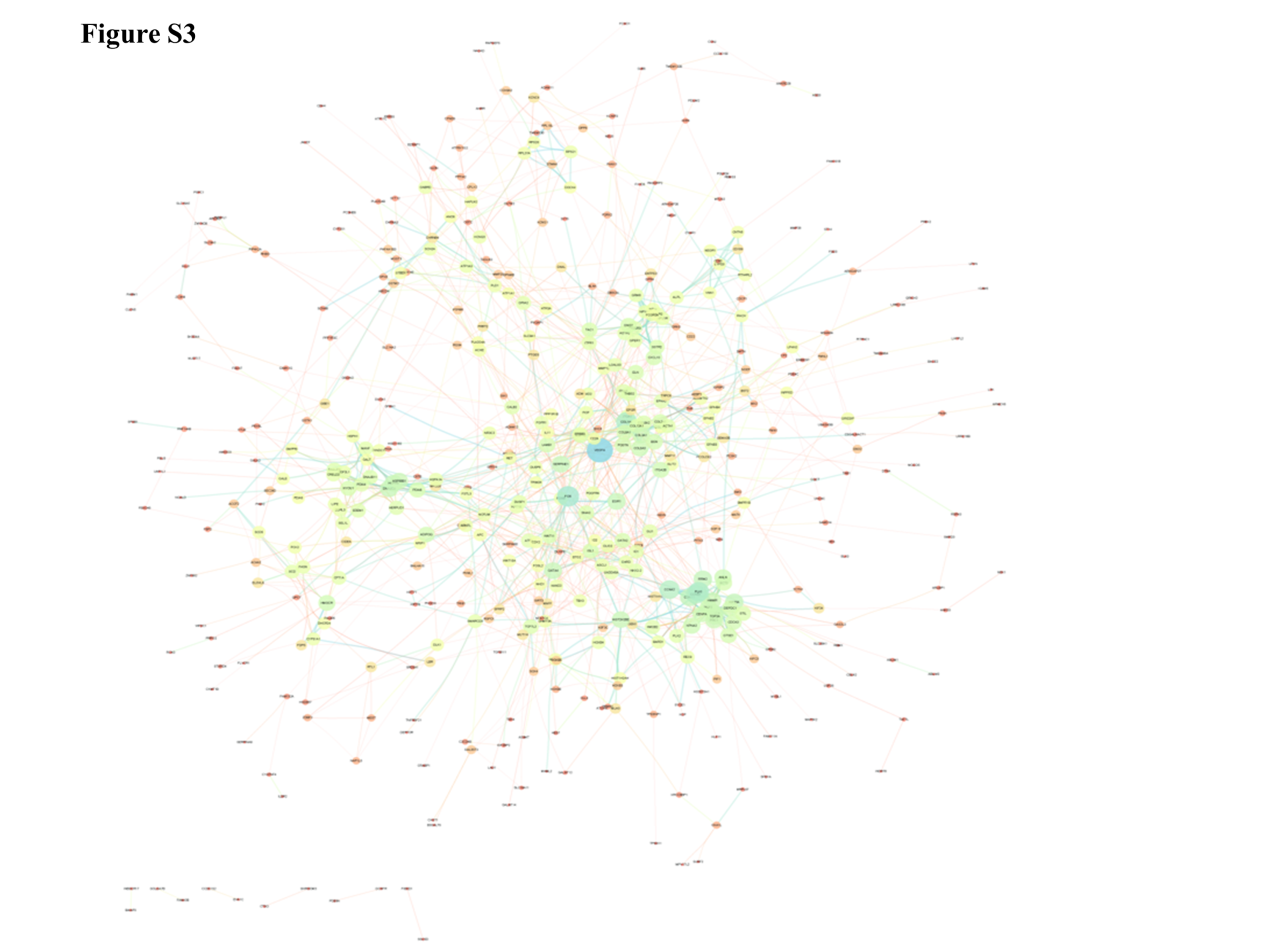

Supplement: Supplementary Materials — Figure S1: GO pathway analysis of the DEGs. GO analysis was conducted in groups of QC-OE vs. QC-Ctrl and covered three domains: cellular components, biological process, and molecular function. The top 20 biological processes in which the upregulated (a) or downregulated (b) genes were significantly enriched. The top 20 cellular components in which the upregulated (c) or downregulated (d) genes were significantly enriched. The top 20 molecular functions in which the upregulated (e) or downregulated (f) genes were significantly enriched. Figure S2: KEGG pathway analysis of the DEGs. KEGG analysis was conducted in groups of QC-OE vs. QC-Ctrl. The top 20 signaling pathways in which the upregulated (a) or downregulated (b) genes were significantly enriched. Figure S3: the PPI network of the DEGs. The online biological resource database Search Tool for the Retrieval of Interacting Genes/Proteins (STRING) 7 was used to identify the interactions between known and predicted proteins. The PPI network was constructed with a score > 0.408, and visualized by Cytoscape 3.7.2, a free software package for visualizing, modeling, and analyzing the integration of biomolecular interaction network with high-throughput expression data. The plug-in named Molecular Complex Detection (MCODE) was then used to filter the modules of the PPI network with parameters set as follows: K − core = 2, node score cutoff = 0.2, degree cutoff = 2, and Max depth up to 100. 552 nodes and 1677 edges are displayed (a). Node color represents the degrees of DEGs, in which brighter node colors correspond to higher degrees of DEGs. Node size represents the closeness centrality, in which the node size is proportional to its closeness centrality. Line thickness represents combined score, in which thicker lines indicate a closer relationship between the connected nodes. [file 4154697.f1.docx]
